# Effects of Different Techniques of Malolactic Fermentation Induction on Diacetyl Metabolism and Biosynthesis of Selected Aromatic Esters in Cool-Climate Grape Wines

**DOI:** 10.3390/molecules23102549

**Published:** 2018-10-06

**Authors:** Małgorzata Lasik-Kurdyś, Małgorzata Majcher, Jacek Nowak

**Affiliations:** 1 Department of Fermentation and Biosynthesis, Faculty of Food Science and Nutrition, Poznań University of Life Sciences, Wojska Polskiego 31, 60-624 Poznań, Poland; jacek.nowaktz@up.poznan.pl; 2 Department of Food Chemistry and Instrumental Analysis, Faculty of Food Science and Nutrition, Poznań University of Life Sciences, Wojska Polskiego 31, 60-624 Poznań, Poland; majcherm@up.poznan.pl

**Keywords:** malolactic fermentation, coinoculation, diacetyl, esters, aromatic compounds, grape wine

## Abstract

The effects of different malolactic bacteria fermentation techniques on the bioconversion of aromatic compounds in cool-climate grape wines were examined. During three wine seasons, red and white grape wines were produced using various malolactic fermentation induction techniques: Coinoculation, sequential inoculation, and spontaneous process. Volatile compounds (diacetyl and the products of its metabolism, and selected ethyl fatty acid esters) were extracted by solid phase microextraction. Compounds were identified with a multidimensional gas chromatograph—GC × GC-ToFMS with ZOEX cryogenic (N_2_) modulator. Sensory evaluation of the wines was also performed. It was found that the fermentation-derived metabolites studied were affected by the malolactic bacteria inoculation regime. Quantitatively, ethyl lactate, diethyl succinate, and ethyl acetate dominated as esters with the largest increase in content. The total concentration of ethyl esters was highest for the coinoculation technique, while the highest concentration of diacetyl was noted for the spontaneous technique. Controlled malolactic fermentation, especially using the coinoculation technique, can be proposed as a safe and efficient enological practice for producing quality cool-climate grape wines enriched with fruity, fresh, and floral aromas.

## 1. Introduction 

Malolactic fermentation (MLF) is a secondary fermentation that takes place after alcoholic fermentation in the process of grape wine production. It is used in the majority of red wines, some white wines, and generally for wines with enhanced acidity. The process is conducted by malolactic bacteria (MLB), most often strains of *Oenococcus oeni*, and involves the decarboxylation of l-malic acid into l-lactic acid. As a result of this bioconversion, a noticeable reduction in the total acidity of the wine can be achieved. The MLB utilize not only malic acid, but also the residual sugars left by yeast after the alcoholic fermentation. This reduces the potential carbon source for spoilage microbiota, and increases microbial stabilization of the wine [1,2,3,4,5]. 

MLF is also a process known to modify the aroma profile of the wine through biosynthesis or bioconversion of flavor-active compounds. Some authors suggest that MLF enhances fruity notes and buttery aromas, while reduces vegetative green and grassy aromas [4,6]. Other researchers have postulated that MLF results in a creamier palate, less fruit intensity, and more nutty, vanilla, toasty butter, and wet leather aromas [7].

A range of factors, including grape cultivar, the bacterial culture used, and the conditions of vinification, have been described as having an effect on the character of wines treated by MLF [8,9]. However, little is known about the effect of different timings of malolactic bacteria inoculation on wine aroma modification. To our knowledge, only a few research groups [9,10,11,12] have compared different MLB inoculation timings and determined their effect on the differentiation of the quality and quantity of the aroma compounds. However, none of these authors examined the spontaneous variant of MLF, which involves a risk of producing undesirable aroma compounds in wines. 

Our research focused on selected aroma compounds (diacetyl and its metabolic products, as well as selected ethyl fatty acid esters) synthesized during malolactic fermentation in white and red grape wines produced by three different methods of inoculation: (1) Coinoculation (COI), where the yeast and bacteria were inoculated at the same time; (2) sequential inoculation (SEQI), where malolactic fermentation was induced at the end of alcoholic fermentation; and (3) spontaneous malolactic fermentation (SPONT), where MLB inoculation was not performed. This is the first study which attempts to evaluate the effects of different timings of MLB inoculation (including the spontaneous process which involves a significant risk of producing undesirable compounds) on the bioconversion of aromatic compounds in red and white grape wines. The experiment was performed during three wine seasons.

## 2. Results and Discussion

### 2.1. Chemical Characteristics of Wines and Dynamics of Malolactic Fermentation

The wines produced through different techniques of malolactic fermentation induction were characterised three months after bottling (Table 1). Almost 90% of the sugars (glucose and fructose) were utilised during the first week of the vinification process. Thus, for the sequential inoculation variant in which MLF was induced at the end of alcoholic fermentation (AF), bacteria inoculation was applied after one week. The final concentration of residual sugars ranged from 2.05 to 5.08 g/L, therefore the obtained wines were classified as dry. No significant influence of MLF on the final ethanol concentration was noted in all the wines. The volatile acidity did not exceed 0.85 g/L (expressed as acetic acid), which is in consonance with the standards of good-quality grape wines.

The course of malolactic fermentation was monitored for all the performed vinification processes, as presented in our previous study [13]. The greatest dynamics of MLF were registered during the first month of vinification. In the second and third months, the amount of malic acid still decreased, but at a much slower rate. Beyond three months, a stabilization of the process was observed, where malic acid reduction for the most part was not significantly important. Based on the dynamics and effectiveness of malic acid reduction, the MLF induction techniques followed the order: COI > SEQI > SPONT > AF [13]. The highest dynamics of malic acid reduction were observed for the coinoculation variant, independent of the vine variety or season. Whereas, much lower efficiency was noted for variants with no bacteria inoculation—alcoholic fermentation and spontaneous MLF, as described in detail in our previous study [13]. No complete reduction of malic acid was reached in any of the wines. The lowest final concentration of malic acid was noted for coinoculation cases.

### 2.2. Ethyl Fatty Acid Esters

The synthesis and hydrolysis of esters during the malolactic fermentation of wines has been described by many research groups, but there is a disagreement concerning the influence of this secondary fermentation action on the final concentration of esters. Most authors indicate that there is a significant enhancement in the ester content of wines that have undergone MLF [8,10,11,12,14,15,16,17,18,19], but other researchers have presented a decreasing trend [20,21,22]. Malolactic bacteria strain selection has also been described as an important factor that determines the final concentrations of esters [12,14,16,17].

Ethyl fatty acid esters are compounds considered to be of primary importance for the aroma of wines. The compounds analyzed include ethyl lactate, diethyl succinate, ethyl propanoate, ethyl hexanoate, ethyl octanoate, and acetate esters (ethyl acetate, isoamyl acetate, and 2-phenethyl acetate).

Ethyl lactate, one of the most characteristic aromatic compounds produced during malolactic fermentation, is synthetized in the course of the esterification of ethanol (produced by yeast during alcoholic fermentation) and lactate (produced by malolactic bacteria during malolactic fermentation). When the malolactic process takes place, the concentration of ethyl lactate progressively increases. This is beneficial to the wine bouquet due to its fruity, buttery, and creamy aromas, and it also contributes to the sensations of roundness in the mouth [16,23]. Some authors suggest that the insensitivity of ethyl lactate biosynthesis depends on the strain of *O. oeni* used [9,15,17,24]. On the other hand, some other authors did not register any dependence of ethyl lactate biosynthesis on the bacteria strain [12].

In our study, the concentration of ethyl lactate after alcoholic fermentation falls in the 8.54–14.44 g/L range (Table 2), and this significantly increased as a result of malolactic fermentation. The highest concentration of ethyl lactate was registered in the coinoculation technique (132.57–173.76 mg/L). After sequential inoculation of MLB and spontaneous MLF, the concentrations of ethyl lactate were significantly lower (41.64–115.63 mg/L). However, this value was several times higher than without MLF (Table 2). The ethyl lactate biosynthesis was thus noted as strongly influenced by the method of malolactic fermentation induction. For the coinoculation variant (with the highest dynamics and effectiveness of malolactic bioconversion) [13], the highest ethyl lactate concentration in the final wine was obtained (Table 2). Whereas, in the variants with lower MLF rate, a lower ethyl lactate synthesis was noted.

Literature studies have reported a wide range of ethyl lactate concentrations in wines that have undergone MLF. Values significantly higher than ours were described by Knoll et al. (up to 440 mg/L) [17], Pozo-Bayon et al. (up to 235 mg/L) [15], and Valade and Laurent (up to 190 mg/L) [25]. Our results are similar to those of Lloret et al., who found ethyl lactate in wines after MLF at concentrations ranging from 90 to 150 mg/L [26]. On the other hand, Fleet [27] and Maicas et al. [14] described standard concentrations for ethyl lactate in red wines of up to 50 mg/L. The aroma threshold for ethyl lactate has been determined to be 110 mg/L [26]. In line with this, ethyl lactate was discernible in our experiment only in wines in which the yeast and MLB had been coinoculated. 

Diethyl succinate is another volatile compound that contributes to wine aroma. Succinic acid (a by-product of microbial α-ketoglutarate metabolism) is esterified to diethyl succinate, which brings fruity melon notes. This compound occurs naturally in apples, grapes, and cocoa. Its odor threshold has been set at 1.2 mg/L [28]. When only alcoholic fermentation was performed in our experiments, the concentration of this compound was found to be under the threshold value (up to 0.68 mg/L). In the wines that underwent MLF, the concentration of diethyl succinate was in the 0.49–2.63 mg/L range (Table 2). Analyzing the influence of different malolactic bacteria inoculation techniques on diethyl succinate concentration, a similar trend to ethyl lactate was noted. The highest concentration of diethyl succinate was always observed in the case of coinoculated wines (1.14–2.63 mg/L) (Table 2), in which the highest rate of MLF was observed [13]. Whereas, in the sequential and spontaneous malolactic process, in which the deacidification rate were significantly lower [13], the concentration of diethyl succinate was also respectively decreased. Similar observations have been described by Knoll et al. [9], who noted that sequential inoculations resulted in lower concentrations of ethyl lactate and diethyl succinate than in the case of coinoculation.

With regard to other esters, no effects of the malolactic process on the biosynthesis of ethyl hexanoate (fruity, strawberry, and green apple aromas) or ethyl octanoate (fruity, sweet, banana, and pear aromas) were observed. All wines that underwent malolactic fermentation showed a significantly enhanced concentration of ethyl propanoate (pineapple aroma) (Table 2). Different observations have been described by Knoll et al. [9], in which a decrease in the concentrations of ethyl hexanoate and ethyl octanoate were reported. Similar to our findings, there was an increase in ethyl propanoate concentration after MLF. Summarizing fluctuations in ethyl fatty acid esters, ethyl lactate and diethyl succinate quantitatively dominated the process, and were the esters showing the greatest increase in concentration. Similar to our results, a significant increase in the concentration of ethyl lactate, ethyl propanoate, and diethyl succinate was also observed after MLF during vinification of Riesling wine [9,17,29], Aglianico wine [16], and Tempranillo and Merlot wine [12,15]. 

A second important group of wine esters is the acetate esters group. For this study, we selected: Ethyl acetate, isoamyl acetate, and 2-phenethyl acetate.

Ethyl acetate is synthesized from ethanol and acetic acid, which are key metabolites in the vinification process. When its concentration does not exceed 100 mg/L, a desirable and fruity aroma enriches the wine. Its presence in higher concentrations leads to solvent, nail, varnish, and chemical aromas [4,14,30]. Isoamyl acetate introduces pleasant fruity notes (mostly banana) to wine aroma profiles. This ester is formed from isoamyl alcohol and acetic acid, intermediate metabolites of alcoholic and malolactic fermentation. The highest concentrations of both ethyl acetate (88.13–119.33 mg/L) and isoamyl acetate (0.76–0.94 mg/L) were always noted with spontaneous MLF (Table 3). This may be due to the notably higher concentrations of volatile acidity (as acetic acid) for this variant of vinification, as described in our previous research [13]. The different inoculation methods significantly affected the final concentration of ethyl acetate and isoamyl acetate in the white and red wines, but the concentrations did not exceed 86.87 and 0.66 mg/L, respectively (Table 3). Maicas et al. [14] found additionally that the production of these compounds was dependent on the MLB strain. In their study, the concentrations after MLF varied from 36.94 to 216.04 mg/L of ethyl acetate, and from 0.25 to 0.68 mg/L of isoamyl acetate. 

2-phenethyl acetate is a volatile metabolite which gives wine floral, honey, and raspberry aromas. In all the examined wines, malolactic bioconversion significantly increased (by almost a factor of two) its concentration over that of the control process (only alcoholic fermentation). However, no effect of inoculation variant on the biosynthesis of 2-phenethyl acetate was noted. Knoll et al. [9] found that wines with sequential MLF had lower concentrations of acetate esters and ethyl esters than coinoculated wines. Ester concentrations were also affected by the bacteria strain used.

### 2.3. Diacetyl and Its Metabolic Products

Diacetyl (2,3-butanedione) is produced by yeast during alcoholic fermentation by a pathway linked to the amino acid metabolism. At the end of alcoholic fermentation, diacetyl is reduced by diacetyl reductase to acetoin (3-hydroxy-2-butanone) and 2,3-butanediol. At this stage of the winemaking process, the concentration of diacetyl thus has no olfactive effect. Significantly higher amounts of diacetyl can be produced during malolactic fermentation as an intermediate product in citric acid metabolism [4,8,14,30,31]. In the course of the MLB carbohydrate metabolism pathway, pyruvate is reduced to lactate. However, when the concentration of residual sugars is too low, citric acid begins to be utilized as a carbon source, and additional pyruvate is synthesized. This pyruvate is then a precursor in the process of diacetyl production. The utilization of citric acid starts simultaneously with malic acid degradation, but it is a very slow process. It is particularly observed in states of sugar deficiency [31]. Subsequently, because of its low chemical stability, diacetyl can be easily transformed into acetoin and 2,3-butanediol [4,7,32].

Malolactic fermentation is thus highly recommended to allow diacetyl to enter into the wine aroma profile. During the secondary fermentation, diacetyl, acetoin, and 2,3-butanediol appear in different concentrations, which has a direct effect on wine aroma. Each of these compounds has a different odor detection threshold. Acetoin and 2,3-butanediol have significantly higher threshold values of perceptibility than diacetyl, at an average of 150 and 600 mg/L, respectively [7,33]. The aroma detection threshold value for diacetyl depends on the type and style of wine. In general, for good quality young red wines it ranges from about 0.2 to about 1.84 mg/L, and for aged red wines from 1.25 to 3.39 mg/L [33].

**Figure 1:**
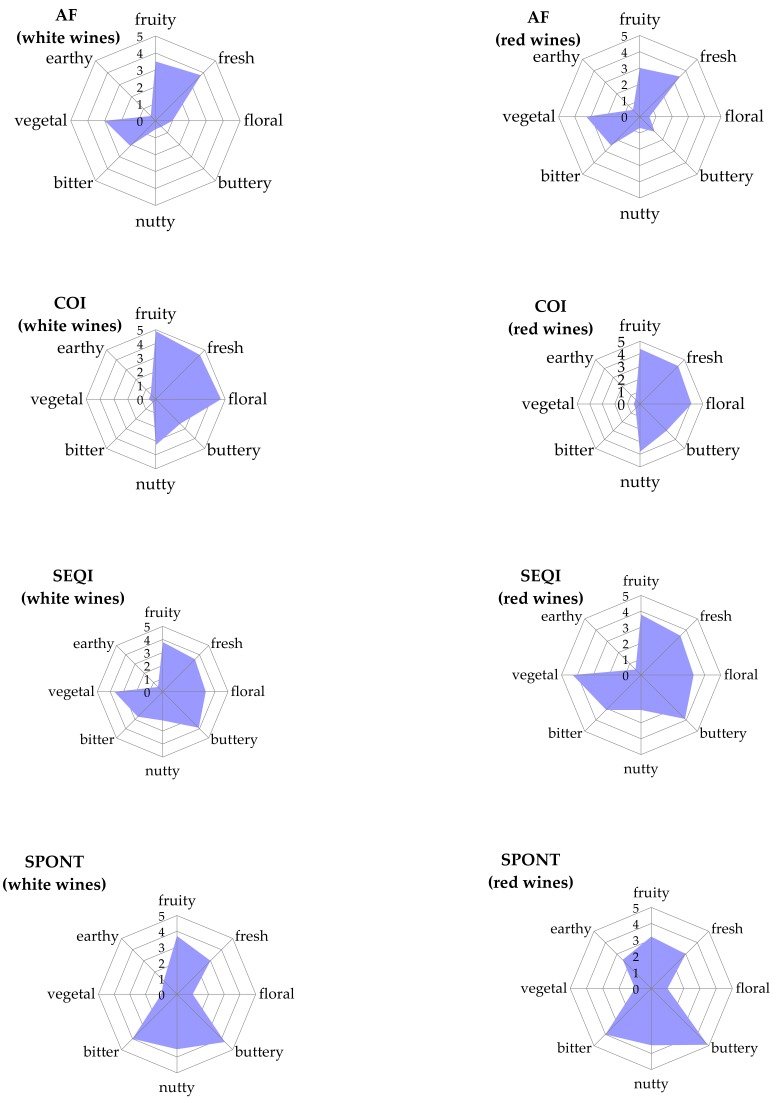
Descriptive sensory evaluation of white and red wines produced by different methods of MLF induction; evaluation was performed three months after bottling. Scale 0–5: 0—lack, 1—very light, 2—light, 3—noticeably, 4—intensive, 5—very intensive sensibility (value 5 is the most desirable for fruity, fresh, and floral aromas, but undesirable and not acceptable for buttery, nutty, bitter, vegetal, and earthy aromas).

The lowest concentration of diacetyl noted in our study occurred in the case of alcoholic fermentation without MLF. The concentration of diacetyl synthesized by the yeasts then ranged from 0.94 to 1.72 mg/L (Table 4), being too low to have an impact on the wine aroma (Figure 1). A significantly higher concentration of diacetyl was noted with malolactic fermentation. In the coinoculation variant this ranged from 2.19 to 4.06 mg/L, and in the sequential inoculation it varied from 3.42 to 5.91 mg/L. For these wines, light, pleasant buttery and nutty aromas were perceptible (Figure 1). The highest concentration of diacetyl was observed for the spontaneous malolactic fermentation (7.44 to 9.22 mg/L); this was characterized as an intensive and unacceptable buttery aroma.

**Figure 2:**
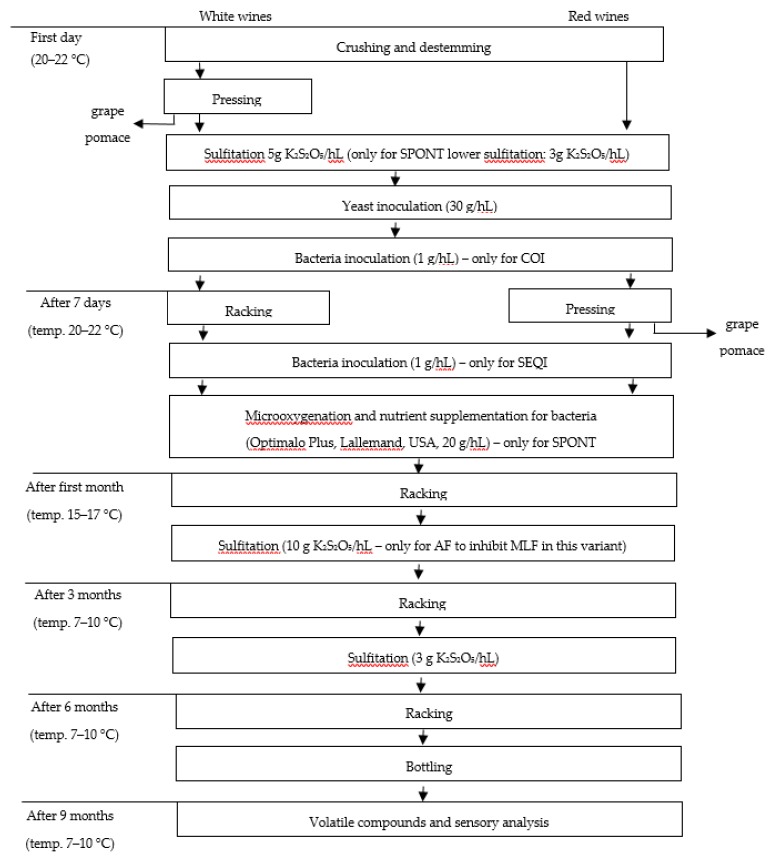
Flowchart of white and red wine production [13].

According to Lerm et al. [34], the accumulation of diacetyl and acetoin depends on the dynamics of malolactic fermentation. The higher the MLF rate, the lower the concentration of diacetyl and acetoin. In our study, the dynamics of MLF followed the order: COI < SEQI < SPONT [13]. This explains the reason for a lesser concentration of diacetyl in the case of coinoculation, and higher concentration for the SEQI and SPONT variants, in which the dynamics of the MLF process were significantly lower. The presence of oxygen during MLF can also affect the diacetyl content in wine. This is directly associated with the oxidation of α-acetolactate to diacetyl [30,34]. In our study, micro-oxygenation was performed to support the initiation of spontaneous MLF (Figure 2) [13]. The additional amount of oxygen present during the vinification process could thus also have led to the significantly higher concentration of diacetyl in this variant (Table 4). 

The products of diacetyl degradation—acetoin (3-hydroxy-2-butanone) and 2,3-butanediol—were also evaluated. In all the wines, the lowest levels of both compounds were noted when only alcoholic fermentation was performed; this concentration ranged from 0.73 to 1.62 mg/L of acetoin, and from 165 to 288 mg/L of 2,3-butanediol (Table 4). Similarly, as in the case of diacetyl, MLF significantly increased the concentration of both metabolites. The highest concentrations were noted for the spontaneous process, in which the biosynthesis yield was as high as 8.79–12.52 mg/L of acetoin, and 652–806 mg/L of 2,3-butanediol. Coinoculation resulted in significantly lower values of both metabolites than sequential inoculation. According to Francis and Newton [33], and Bartowsky and Henschke [7], acetoin levels remained under the sensory threshold, but 2,3-butanediol reached a concentration of sensory significance for wine (over 600 mg/dm^3^), though only in the spontaneous variants. This was reflected in the sensory evaluation of the produced wines (Figure 1). Generally, 2,3-butanediol is not expected to affect the sensory qualities of wine appreciably [35], but we did note a bitter taste in these variants. Some authors have also described very low or undetectable levels of diacetyl in wines that had undergone MLF [36]. They suggest that this may be a result of the enzymatic reduction of diacetyl to 2,3-butanediol. Acetoin, the other intermediate metabolite of diacetyl involved in the same metabolic pathway, is also reduced to 2,3-butanediol. This may explain the high levels of 2,3-butanediol found in our wines.

### 2.4. Sensory Evaluation

The sensory evaluation indicated that the timing and method of MLB inoculation significantly affected the taste and aroma of the wines. In general, malolactic fermentation diversified the wine aroma profile (Figure 1). The coinoculated wines were noted to be higher in fruity, fresh, and floral sensations than the wines which had used sequential MLF. The spontaneous process was perceived as producing wines with more buttery and bitter notes. Pleasant balanced buttery and nutty aromas were also found in the coinoculated wines. The wines after sequential and spontaneous processes had no nutty aromas, but strong buttery aromas were noted instead.

## 3. Materials and Methods

### 3.1. Microorganisms

*Saccharomyces cerevisiae* yeast (Lalvin EC-118, Lallemand, Petaluma, CA, USA) and *Oenococcus oeni* bacteria (Lalvin VP41, Lallemand, Petaluma, CA, USA) were used in the experiments. Rehydration protocols were followed as per the producer’s instructions.

### 3.2. Grape Variety 

During three wine seasons, two white varieties, Chardonnay 2009 and Kerling 2010, and two red varieties, Pinot noir 2009 and 2012, and Rondo 2012, were used to produce the experimental wines. The grapes were obtained from Mierzecin Vineyard in Poland. Typically for grapes from cool-climate countries, the musts were characterized with enhanced total acidity (9.38–12.14 g/L, as tartaric acid) and low pH (3.19–3.64) (Table 5) [13], which significantly singularize them from others studied and described in the literature [9,10,11,12,37,38]. 

### 3.3. Parameters of the Vinification Process

The wines were produced on a laboratory scale in 15-liter glass containers. Four different variants of the vinification process were performed: (1) Alcoholic fermentation only, as a control (AF); (2) coinoculation (COI), where the yeast and bacteria were inoculated at the same time; (3) sequential inoculation (SEQI), where malolactic fermentation was induced at the end of alcoholic fermentation; and (4) spontaneous malolactic fermentation (SPONT), where we did not perform MLB inoculation. The consecutive steps of the vinification process are presented in Figure 2 as a flowchart, and follow those of our previous study [13]. 

The process commenced with the inoculation of yeast in the first day for all variants. The timing of malolactic bacteria inoculation was dependent on the variant. This was done on the first day (together with yeasts) in COI samples, and on the seventh day of winemaking for SEQI samples. To avoid spontaneous MLF in the AF variant, an additional sulfitation process was performed after one month of vinification (10 g/hL of K_2_S_2_O_5_). In the SPONT samples, MLF was induced by micro-oxygenation, supplementation with bacteria nutrients, and lower sulfitation. No malolactic bacteria starter culture was added in this case. The winemaking process lasted six months. Next, after bottling, the wines were subjected to maturation (temp. 7–10 °C) for three more months. Finally, after nine months, the obtained wines were submitted for chemical and sensory analysis. The samples were stored at 7–10 °C, without freezing.

### 3.4. Analysis of Volatile Compounds 

The chemical analysis of volatile compounds was performed for all the wines of the three seasons at the same time (3 months after bottling). Before analysis, samples were only filtered with 0.45-µm Millipore filters.

The volatiles were extracted by solid phase microextraction using a 2 cm DVB/CAR/PDMS fiber (Supelco) with a CTC Combipal autosampler (Agilent Technologies). For each analysis, 10 mL samples of wine were placed into 20 mL vials, spiked with an internal standard ([^2^H_8_]-naphthalene), sealed with PTFE/silicon septa caps, and incubated for 2 min at 50 °C prior to extraction. Compounds were extracted from the headspace at 50 °C for 35 min. Compounds were identified using multidimensional gas GCxGC-ToFMS chromatography with a ZOEX cryogenic (N_2_) modulator (Pegasus IV, LECO, St. Joseph, MI, USA). The GC was equipped with a DB-5 column (30 m × 0.25 mm × 0.25 μm), and had a Supelcowax 10 (1 m × 0.1 mm × 0.1 μm) as a second column, with a helium flow rate of 0.8 mL/min. For the two-dimensional analysis, the modulation time was optimized and set at 3 seconds, and the mass spectra were collected at a rate of 150 scans/s. The transfer line was heated to 280 °C, and the ion source was heated to 220 °C. The injector temperature was set to 280 °C for the DVB/CAR/PDMS fiber. During the injection, the fiber was maintained for 5 min in the splitless mode, and then for 1 min in the split mode (20:1). Identification of volatiles was performed by comparison of the retention indices and mass spectra of eluting compounds to those of the NIST 05 library match. The calculation was done using Chroma TOF software (version 4.23) upgraded with additional post data processing Statistical Compare software (LECO, St. Joseph, MI, USA) for calculation of the Fisher ratio. Semiquantification of the volatile compounds was performed using the internal standard; thus, they do not represent the absolute amount of the compound present in the wine samples, but were instead calculated and used to observe the differences between the wine samples. Each measurement was repeated three times.

### 3.5. Chemical Analysis of Must and Wine

The chemical analysis of the wines was performed for all the wines of the three seasons at the same time (3 months after bottling). Before analysis, samples were only filtered with 0.45-µm Millipore filters. The soluble solids (°Bx) were evaluated by refractometer. To control the chemical parameters, total acidity was measured according to OIV-MA-AS313-01, pH according to OIV-MA-AS313-15, and volatile acidity according to OIV-MA-AS-313-02 [39]. The concentration of glucose, fructose, malic acid, citric acid, and ethanol was measured using HPLC (Waters Alliance 2695 combined with BioRad Aminex HPX-87H column, 300 × 7.8 mm and RI detector; measurement conditions: 1.5 mM H_2_SO_4_ as eluent, flow rate 0.4 mL/min, column temp. 50 °C, and detector temp. 45 °C). 

### 3.6. Sensory Evaluation

The sensory evaluation was performed for all the wines of the three seasons at the same time (3 months after bottling). Sensory analysis was undertaken in order to evaluate the differences between the wines obtained with different inoculation techniques. The sensory panel members included 120 people between 24 and 55 years old. They evaluated the wine samples using a 0–5 point scale (0 = very low discernible aroma; 5 = very intensive discernible aroma). The applied serving temperature was kept at 13–15 °C for white wines, and at 17–18 °C for red wines.

### 3.7. Statistical Analysis

All data are presented as the mean value of at least three repetitions ± standard deviation. Statistical data analysis was performed using analysis of variance (ANOVA) in Statistica V.8 (Statsoft Inc., Tulsa, OK, USA). Tukey’s test was used for significantly different samples (*p* < 0.05). 

## 4. Conclusions

The fermentation-derived metabolites examined in this study were affected by the malolactic bacteria inoculation technique. The total concentration of the analyzed ethyl esters was highest for the coinoculation case. Quantitatively, ethyl lactate, diethyl succinate, and ethyl acetate dominated as the esters that showed the greatest increase in concentration. An excess of diacetyl, perceived as a serious danger of MLF which can negatively affect the quality of wine, was noted only for the spontaneous processes. Whereas, coinoculation was a treatment with unbeatably balanced nutty notes. 

The present investigations highlighted that controlled malolactic fermentation, and especially the coinoculation technique, can be proposed as a safe and efficient enological practice for producing quality grape wines. Our results clearly indicate that simultaneous inoculation of yeast and bacteria offer not only dynamic deacidification of low pH grape wines [13], but also modify qualitatively and quantitatively the profile of volatile compounds, enriching the cool-climate and low aromatic wines with fruity, fresh, and floral aromas.

## Figures and Tables

**Table 1:** Chemical characteristics of wines produced by different methods of MLF induction; parameters were evaluated three months after bottling.

		pH	Total Acidity (g/L) *	Volatile Acidity (g/L) **	Ethanol (% *v*/*v*)	Residual Sugars (g/L)	
						After 1 Week ***	3 Months After Bottling
	Chardonnay						
White wines	AF	3.35 ± 0.02 ^a^	7.15 ± 0.12 ^c^	0.75 ± 0.03 ^b^	12.5 ± 0.12 ^a^	17.53 ± 0.51 ^b^	5.08 ± 0.17 ^c^
	COI	3.65 ± 0.04 ^c^	5.75 ± 0.09 ^a^	0.69 ± 0.04 ^a^	12.4 ± 0.16 ^a^	11.12 ± 0.55 ^a^	2.05 ± 0.53 ^a^
	SEQI	3.49 ± 0.06 ^b^	6.34 ± 0.11 ^b^	0.70 ± 0.01 ^a^	12.5 ± 0.19 ^a^	19.23 ± 0.42 ^c^	2.52 ± 0.23 ^a^
	SPONT	3.38 ± 0.04 ^a^	7.03 ± 0.14 ^c^	0.85 ± 0.03 ^c^	12.4 ± 0.13 ^a^	18.92 ± 0.31 ^c^	4.52 ± 0.41 ^b^
	Kerling						
	AF	3.26 ± 0.02 ^a^	7.35 ± 0.12 ^c^	0.66 ± 0.05 ^c^	11.2 ± 0.11 ^a^	16.71 ± 0.26 ^c^	4.71 ± 0.09 ^d^
	COI	3.58 ± 0.02 ^d^	6.12 ± 0.09 ^a^	0.41 ± 0.06 ^a^	11.1 ± 0.08 ^a^	14.39 ± 0.74 ^b^	2.09 ± 0.12 ^a^
	SEQI	3.42 ± 0.05 ^c^	7.17 ± 0.14 ^b^	0.49 ± 0.03 ^b^	11.0 ± 0.06 ^a^	12.43 ± 0.41 ^a^	2.41 ± 0.11 ^b^
	SPONT	3.37 ± 0.07 ^b^	7.19 ± 0.11 ^b^	0.77 ± 0.06 ^d^	11.2 ±0.13 ^a^	17.38 ± 0.55 ^d^	4.05 ± 0.18 ^c^
Red wines	Pinot noir A						
	AF	3.71 ± 0.05 ^a^	5.69 ± 0.07 ^b^	0.44 ± 0.04 ^b^	13.2 ± 0.12 ^a^	18.18 ± 0.42 ^d^	4.78 ± 0.15 ^b^
	COI	4.00 ± 0.09 ^d^	5.06 ± 0.09 ^a^	0.53 ± 0.07 ^c^	13.1 ± 0.16 ^a^	12.53 ± 0.75 ^a^	2.14 ± 0.06 ^c^
	SEQI	3.90 ± 0.06 ^c^	5.77 ± 0.11 ^c^	0.39 ± 0.06 ^a^	13.3 ± 0.22 ^a^	16.87 ± 0.39 ^b^	2.37 ± 0.08 ^a^
	SPONT	3.81 ± 0.03 ^b^	5.52 ± 0.09 ^b^	0.69 ± 0.03 ^d^	13.3 ± 0.18 ^a^	17.94 ± 0.46 ^c^	3.94 ± 0.11 ^d^
	Pinot noir B						
	AF	3.52 ± 0.04 ^a^	6.48 ± 0.16 ^b^	0.41 ± 0.04 ^a^	12.1 ± 0.11 ^a^	19.62 ± 0.38 ^d^	5.08 ± 0.09 ^d^
	COI	3.81 ± 0.05 ^c^	5.81 ± 0.09 ^a^	0.42 ± 0.03 ^a^	12.1 ± 0.09 ^a^	13.93 ± 0.25 ^a^	3.12 ± 0.13 ^a^
	SEQI	3.75 ± 0.02 ^b^	5.94 ± 0.07 ^a^	0.47 ± 0.09 ^a^	12.0 ± 0.13 ^a^	17.12 ± 0.74 ^b^	3.80 ± 0.11 ^b^
	SPONT	3.58 ± 0.08 ^a^	6.44 ± 0.11 ^b^	0.66 ± 0.05 ^b^	12.0 ± 0.17 ^a^	18.27 ± 0.71 ^c^	4.44 ± 0.16 ^c^
	Rondo						
	AF	3.30 ± 0.03 ^a^	6.92 ± 0.12 ^c^	0.49 ± 0.04 ^a^	12.2 ± 0.14 ^a^	16.48 ± 0.52 ^d^	4.45 ± 0.19 ^c^
	COI	3.66 ± 0.05 ^d^	5.37 ± 0.13 ^a^	0.59 ± 0.04 ^b^	12.2 ± 0.12 ^a^	12.69 ± 0.84 ^a^	3.24 ± 0.12 ^a^
	SEQI	3.53 ± 0.03 ^c^	6.52 ± 0.06 ^b^	0.56 ± 0.09 ^b^	12.0 ± 0.06 ^a^	14.36 ± 0.51 ^b^	4.00 ± 0.15 ^b^
	SPONT	3.47 ± 0.07 ^b^	6.88 ± 0.12 ^c^	0.69 ± 0.07 ^c^	12.1 ± 0.26 ^a^	15.73 ± 0.73 ^c^	5.03 ± 0.13 ^d^

The data are the mean of triplicates ± SD; AF—only alcoholic fermentation; COI—coinoculation of yeast and bacteria; SEQI—sequential inoculation of yeast and bacteria; SPONT—spontaneous malolactic fermentation; * as tartaric acid; ** as acetic acid; *** values of residual sugar concentration after 1 week are presented to explain why bacteria inoculation in SEQI was performed after 7 days; ^a, b, c, d^—denotes statistically significant differences (*p* < 0.05) between the different inoculation techniques.

**Table 2:** Concentrations (mg/L) of esters in white and red grape wines produced by different methods of MLF induction; parameters were evaluated three months after bottling

		Ethyl Lactate	Ethyl Propanoate	Ethyl Hexanoate	Ethyl Octanoate	Diethyl Succinate
White wine	Chardonnay					
	AF	8.54 ± 0.31 ^d^	0.31 ± 0.03 ^b^	0.83 ± 0.07 ^a^	1.17 ± 0.09 ^a^	0.68 ± 0.01 ^d^
	COI	134.97 ± 3.93 ^a^	0.36 ± 0.05 ^a^	0.86 ± 0.04 ^a^	1.11 ± 0.09 ^a^	2.63 ± 0.02 ^a^
	SEQ	115.63 ± 5.59 ^b^	0.42 ± 0.04 ^a^	0.79 ± 0.09 ^a^	1.14 ± 0.04 ^a^	1.42 ± 0.06 ^b^
	SPONT	72.43 ± 2.64 ^c^	0.39 ± 0.05 ^a^	0.81 ± 0.05 ^a^	1.12 ± 0.06 ^a^	0.89 ± 0.03 ^c^
	Kerling					
	AF	8.62 ± 0.68 ^d^	0.14 ± 0.04 ^b^	0.53 ± 0.03 ^a^	0.88 ± 0.05 ^a^	0.32 ± 0.05 ^d^
	COI	132.57 ± 4.04 ^a^	0.27 ± 0.07 ^a^	0.57 ± 0.05 ^a^	0.93 ± 0.04 ^a^	1.14 ± 0.03 ^a^
	SEQI	111.66 ± 5.84 ^b^	0.23 ± 0.03 ^a^	0.53 ± 0.04 ^a^	0.89 ± 0.06 ^a^	0.55 ± 0.05 ^b^
	SPONT	71.04 ± 3.61 ^c^	0.21 ± 0.04 ^a^	0.55 ± 0.04 ^a^	0.85 ± 0.08 ^a^	0.49 ± 0.05 ^c^
Red wine	Pinot noir A					
	AF	14.44 ± 0.86 ^d^	0.51 ± 0.03 ^b^	0.74 ± 0.06 ^a^	1.23 ± 0.04 ^b^	0.54 ± 0.03 ^d^
	COI	151.25 ± 4.23 ^a^	0.73 ± 0.05 ^a^	0.77 ± 0.07 ^a^	1.31 ± 0.03 ^a^	2.07 ± 0.09 ^a^
	SEQI	97.32 ± 6.59 ^b^	0.77 ± 0.03 ^a^	0.74 ± 0.05 ^a^	1.26 ± 0.02 ^b^	1.32 ± 0.07 ^b^
	SPONT	64.97 ± 4.44 ^c^	0.74 ± 0.03 ^a^	0.71 ± 0.05 ^a^	1.22 ± 0.05 ^b^	0.76 ± 0.06 ^c^
	Pinot noir B					
	AF	11.43 ±0.41 ^d^	0.39 ± 0.03 ^b^	0.66 ± 0.08 ^a^	1.08 ± 0.06 ^a^	0.47 ± 0.03 ^d^
	COI	173.76 ± 5.72 ^a^	0.62 ± 0.03 ^a^	0.71 ± 0.04 ^a^	1.14 ± 0.08 ^a^	1.96 ± 0.07 ^a^
	SEQI	91.26 ± 3.66 ^b^	0.60 ± 0.04 ^a^	0.68 ± 0.05 ^a^	1.09 ± 0.05 ^a^	0.88 ± 0.05 ^b^
	SPONT	65.33 ± 3.43 ^c^	0.61 ± 0.03 ^a^	0.65 ± 0.06 ^a^	1.06 ± 0.09 ^a^	0.61 ± 0.07 ^c^
	Rondo					
	AF	9.01 ± 0.56 ^d^	0.27 ± 0.03 ^b^	0.64 ± 0.03 ^a^	0.94 ± 0.05 ^a^	0.51 ± 0.06 ^d^
	COI	137.41 ± 6.92 ^a^	0.34 ± 0.04 ^a^	0.69 ± 0.04 ^a^	0.99 ± 0.04 ^a^	1.88 ± 0.11 ^a^
	SEQI	84.78 ± 4.47 ^b^	0.38 ± 0.02 ^a^	0.66 ± 0.03 ^a^	0.93 ± 0.07 ^a^	1.12 ± 0.08 ^b^
	SPONT	41.64 ± 4.33 ^c^	0.35 ± 0.03 ^a^	0.62 ± 0.07 ^a^	0.93 ± 0.08 ^a^	0.74 ± 0.06 ^c^

The data are the mean of triplicates ± SD; AF—only alcoholic fermentation; COI—coinoculation of yeast and bacteria; SEQI—sequential inoculation: bacteria at the end of AF; SPONT—spontaneous MLF; ^a, b, c, d^—denotes statistically significant differences (*p* < 0.05) between the different inoculation techniques.

**Table 3:** Concentrations (mg/L) of esters in white and red grape wines produced by different methods of MLF induction; parameters were evaluated three months after bottling

		Ethyl Acetate	Isoamyl Acetate	2-Phenethyl Acetate	Sum of Esters Total/Without Ethyl Lactate and Ethyl Acetate
White wine	Chardonnay				
	AF	63.43 ± 1.27 ^c^	0.56 ± 0.07 ^b^	0.23 ± 0.01 ^b^	75.75/3.78
	COI	51.67 ± 2.03 ^d^	0.42 ± 0.04 ^c^	0.69 ± 0.03 ^a^	192.71/6.07
	SEQI	68.44 ± 3.16 ^b^	0.63 ± 0.08 ^b^	0.62 ± 0.08 ^a^	189.09/5.02
	SPONT	88.41 ± 2.87 ^a^	0.89 ± 0.03 ^a^	0.73 ± 0.05 ^a^	165.67/4.83
	Kerling				
	AF	72.66 ± 3.016 ^b^	0.66 ± 0.02 ^b^	0.33 ± 0.02 ^b^	84.14/2.86
	COI	58.12 ± 5.37 ^d^	0.49 ± 0.04 ^c^	0.49 ± 0.07 ^a^	194.58/3.89
	SEQI	63.27 ± 5.82 ^c^	0.51 ± 0.03 ^c^	0.52 ± 0.03 ^a^	178.16/3.23
	SPONT	110.26 ± 4.93 ^a^	0.94 ± 0.03 ^a^	0.55 ± 0.04 ^a^	184.89/3.59
Red wine	Pinot noir A				
	AF	77.42 ± 3.28 ^c^	0.33 ± 0.05 ^c^	0.45 ± 0.03 ^b^	95.66/3.8
	COI	71.33 ± 6.71 ^c^	0.28 ± 0.06 ^c^	0.92 ± 0.03 ^a^	228.66/6.08
	SEQI	81.58 ± 4.55 ^b^	0.44 ± 0.02 ^b^	0.88 ± 0.04 ^a^	184.31/5.41
	SPONT	94.11 ± 5.19 ^a^	0.76 ± 0.03 ^a^	0.94 ± 0.06 ^a^	164.21/5.13
	Pinot noir B				
	AF	69.31 ± 6.07 ^c^	0.66 ± 0.09 ^b^	0.37 ± 0.03 ^b^	84.37/3.63
	COI	66.93 ± 4.31 ^c^	0.47 ± 0.03 ^c^	0.84 ± 0.07 ^a^	246.43/5.74
	SEQI	75.21 ± 4.89 ^b^	0.58 ± 0.09 ^b^	0.79 ± 0.09 ^a^	171.09/4.62
	SPONT	88.13 ± 5.26 ^a^	0.83 ± 0.04 ^a^	0.87 ± 0.07 ^a^	158.09/4.63
	Rondo				
	AF	79.17 ± 4.11 ^c^	0.31 ± 0.02 ^c^	0.41 ± 0.02 ^b^	91.26/3.08
	COI	78.33 ± 4.85 ^c^	0.44 ± 0.07 ^b^	0.83 ± 0.05 ^a^	220.91/5.17
	SEQI	86.87 ± 3.17 ^b^	0.38 ± 0.06 ^b^	0.81 ± 0.09 ^a^	175.93/4.28
	SPONT	119.33 ± 4.69 ^a^	0.79 ± 0.03 ^a^	0.88 ± 0.05 ^a^	165.28/4.31

The data are the mean of triplicates ± SD; AF—only alcoholic fermentation; COI—coinoculation of yeast and bacteria; SEQI—sequential inoculation: bacteria at the end of AF; SPONT—spontaneous MLF; ^a, b, c, d^—denotes statistically significant differences (*p* < 0.05) between the different inoculation techniques.

**Table 4:** Concentrations (mg/L) of diacetyl, acetoin, and 2,3-butanediol in white and red grape wines produced by different methods of MLF induction; parameters were evaluated three months after bottling.

		2,3-Butanedione (Diacetyl)	3-Hydroxy-2-Butanone (Acetoin)	2,3-Butanediol
White wines	Chardonnay			
	AF	1.13 ± 0.22 ^d^	0.86 ± 0.06 ^d^	254.32 ± 11.34 ^d^
	COI	2.19 ± 0.15 ^c^	3.11 ± 0.39 ^c^	331.56 ± 15.33 ^c^
	SEQI	3.42 ± 0.19 ^b^	5.95 ± 0.26 ^b^	479.93 ± 9.14 ^b^
	SPONT	7.44 ± 0.08 ^a^	8.79 ± 0.42 ^a^	712.42 ± 23.65 ^a^
	Kerling			
	AF	0.94 ± 0.24 ^d^	0.73 ± 0.04 ^d^	165.36 ± 12.75 ^d^
	COI	3.02 ± 0.16 ^c^	4.31 ± 0.22 ^c^	349.62 ± 16.83 ^c^
	SEQI	4.09 ± 0.08 ^b^	6.18 ± 0.42 ^b^	513.88 ± 8.31 ^b^
	SPONT	8.33 ± 0.22 ^a^	11.05 ± 0.98 ^a^	783.63 ± 10.63 ^a^
Red wines	Pinot noir A			
	AF	1.71 ± 0.15 ^d^	1.62 ± 0.17 ^d^	288.97 ± 12.06 ^d^
	COI	3.80 ± 0.19 ^c^	5.11 ± 0.36 ^c^	361.35 ± 9.72 ^c^
	SEQI	5.24 ± 0.16 ^b^	6.94 ± 0.45 ^b^	647.55 ± 17.83 ^b^
	SPONT	9.22 ± 0.23 ^a^	12.39 ± 0.41 ^a^	806.37 ± 14.97 ^a^
	Pinot noir B			
	AF	1.31 ± 0.14 ^d^	1.04 ± 0.12 ^c^	267.42 ± 8.53 ^d^
	COI	4.06 ± 0.09 ^c^	6.48 ± 0.48 ^b^	493.52 ± 11.84 ^c^
	SEQI	5.91 ± 0.11 ^b^	7.17 ± 0.56 ^b^	631.67 ± 17.59 ^b^
	SPONT	8.72 ± 0.15 ^a^	12.52 ± 0.46 ^a^	718.13 ± 13.77 ^a^
	Rondo			
	AF	1.72 ± 0.05 ^d^	1.53 ± 0.14 ^d^	264.94 ± 6.48 ^d^
	COI	3.81 ± 0.09 ^c^	4.97 ± 0.32 ^c^	355.13 ± 15.83 ^c^
	SEQI	5.50 ± 0.13 ^b^	7.03 ± 0.26 ^b^	584.42 ± 18.28 ^b^
	SPONT	8.80 ± 0.07 ^a^	11.43 ± 0.31 ^a^	652.36 ± 15.75 ^a^

The data are the mean of triplicates ± SD; AF—only alcoholic fermentation; COI—coinoculation of yeast and bacteria; SEQI—sequential inoculation: bacteria at the end of AF; SPONT—spontaneous MLF; **^a^**^, b, c, d^—denotes statistically significant differences (*p* < 0.05) between the different inoculation techniques.

**Table 5:** Characteristic of grape must used for the vinification process [13].

	Chardonnay	Kerling	Pinot Noir A	Pinot Noir B	Rondo
°Bx	22.5 ± 0.1	20.5 ± 0.1	24.0 ± 0.2	22.0 ± 0.1	22.0 ± 0.2
Sugars: glucose + fructose (g/L)	193.12 ± 2.11	171.33 ± 2.54	203.54 ± 3.05	185.47 ± 3.17	181.62 ± 2.84
pH	3.31 ± 0.03	3.19 ± 0.03	3.64 ± 0.05	3.41 ± 0.06	3.26 ± 0.07
Total acidity (g/L) *	11.64 ± 0.12	12.14 ± 0.14	9.38 ± 0.09	10.95 ± 0.23	11.52 ± 0.26
Malic acid (g/L)	8.14 ± 0.09	9.5 ± 0.05	6.54 ± 0.07	8.11 ± 0.14	7.83 ± 0.21
Citric acid (g/L)	0.33 ± 0.02	0.41 ± 0.02	0.28 ± 0.02	0.35 ± 0.02	0.31 ± 0.02

* as tartaric acid.
